# Artificial neural networks (ANNs) for modeling efficient factors in predicting pap smear screening behavior change stage

**DOI:** 10.37796/2211-8039.1240

**Published:** 2022-06-01

**Authors:** Elahe Allahyari, Mitra Moodi, Zoya Tahergorabi

**Affiliations:** a Department of Epidemiology and Biostatistics, School of Health, Social Determinants of Health Research Center, Birjand University of Medical Sciences, Birjand, Iran; b Social Determinants of Health Research Center, Birjand University of Medical Sciences, Birjand, Iran; c Medical Toxicology and Drug Abuse Research Center, Birjand University of Medical Sciences, Birjand, Iran

**Keywords:** Artificial neural network, Cervical cancer, Pap smear, Predicting factor, Screening

## Abstract

**Background and objectives:**

Cervical cancer is ranked as the third most prevalent cancer that affects women all over the world and Pap smear seems to be the single most critical intervention to prevent cervical cancer. In the present study, the effects of demographic factors (age, education level, job, income level, marriage age, pregnancy, child number, breastfeeding, and menopause), insurance type, disease history and screening (sono and mammography, breast problem) and cancer information on Pap smear screening and behavior stage of change were investigated and modeled using an artificial neural network model (ANN).

**Materials and methods:**

Data were collected from a descriptive-analytical cross-sectional study. This research was conducted on 1898 female employees of governmental agencies of Birjand, a city located in the east of Iran. The questionnaire consisted of four parts (socioeconomic, reproductive characteristics, information about cervical cancer screening, and stage of change for cervical cancer screening). Multilayer feed-forward back-propagation neural networks were used to detect the patterns between variables using a neural network with 14 inputs and one output. To find out the neural network with the minimum sum of squared errors, we evaluated the performance of all neural networks using varying algorithms and numbers of neurons in the hidden layer. For this purpose, the data collected from 1898 women were analyzed using SPSS-22 software.

**Results:**

In the optimal ANN model, the variables of marriage age, age, breastfeeding, and the number of children were identified as the most significant factors with 18.3, 16.3, 7.3, and 7.3 percent, respectively, whereas the history of cancer, job, pregnancy, and menopause had importance of lower than 5 percent.

**Conclusion:**

Our findings showed that among many associated variables, the marriage age, age, breastfeeding, and the number of children were the most important predictors for the behavioral stage of change. Thus, it seems, focusing on these factors may lead to the adoption of effective programs and policies to improve cervical cancer screening practices in women.

## 1. Introduction

Cancers are the leading cause of death worldwide particularly in women in both high-income countries and middle-income countries [1]. Among the cancers, cervical cancer is one of the most common gynecological malignancies which were relatively well controlled for several decades in many high-income countries however; it remains the leading cause of cancer-related deaths in 42 countries, most of which are low-income and lower-middle-income countries (LMICs) [2]. Approximately 85% of women who were diagnosed with cervical cancer and 88% of those who died from cervical cancer live in LMICs [1]. Based on the World Health Organization (WHO) report, low and middle-income countries have the highest cervical cancer rates due to different factors including irregular doctor visits, low access to health facilities, and unaffordable health care [3]. Based on the International Agency for Research on Cancer (IARC), globally more than 569,000 new cases of cervical cancer are reported, and 311,000 women deaths occur annually due to cervical cancer [4,5].

The most widely accepted, method of cervical cancer screening in the early stages of the disease, which is inexpensive, simple, quick, and relatively painless is the Papanicolaou smear (Pap smear) [6]. Cervical cancer can be prevented through primary, secondary, and tertiary prevention methods [7]. Primary prevention method includes education for having safe sexual practices and human papillomavirus (HPV) vaccination [8]. HPV vaccination is considered as one of the proven approaches to prevent cervical cancer however, in many countries, implementation of HPV vaccination is limited (4). The secondary prevention method involves cervical cancer screening of asymptomatic patients and management of precancerous lesions before they turn into cancer via Pap smear [9,10]. Experiences from the developed world show, broad and effective coverage of screening programs can lead to a reduction in cervical cancer incidences and mortality associated with this cancer [6,11]. The screening programs for cervical cancer in Iran are free of charge and widely accessible in public health centers however, Iranian women commonly avail these cervical cancer screening services resulting in a high rate of death from cervical cancer in Iran [12].

Several epidemiological studies have shown the role of risk factors such as sexually transmitted infections [13], reproductive and behavioral factors [14] in the incidence of cancer which among them, HPV infection is the most important risk factor [15,16]. Also, sociodemographic factors which influence women’s screening practices include age, marital status, education level, employment status, smoking, long-term hormonal contraceptive use, and income level [17,18], which some of them are modifiable variables and can impact the well-being of individuals [19].

Since decision-making processes for Pap smear screening are very complex and women may be in different stages of readiness of adopting a behavior change hence, the transtheoretical model (TTM) of behavior change was used in this study. Also, based on the TTM model, health-seeking behavior is a dynamic process. This model has four basic principles: the stages of change, self-efficacy, decisional balance, and processes of change [20,21].

By modeling the relationships of neurons in the human brain, artificial neural networks (ANN) were identified, predicted, and classified the pattern of association between variables [22]. This type of modeling has been used in psychiatry, medicine, etc. with a lower sum of square error and higher correlation coefficients than conventional models such as the response surface methodology (RSM) [23,24].

Results from a study conducted by Schaffter et al., (2020) on 144,231 mammograms from 85,580 American women and 166,578 examinations from 68,008 Swedish women showed no single artificial intelligence algorithm outperformed the US community radiologist benchmarks [25]. Another study was conducted by Natarajan et al. (2019) on 231 patients for diabetic retinopathy screenings. In this study, the use of an offline artificial intelligence system on a smartphone-enabled screening of referable diabetic retinopathy in remote areas where services of an ophthalmologist are unavailable with sensitivity and specificity of 100.0% and 88.4%, respectively vs. 85.2% and 92.0% with ophthalmologist grading using the same images, respectively [26]. Also, Bhatt et al., (2021) used singular multi-class classification methodology and Progressive Resizing and Transfer Learning on Deep Neural Network models for cervical cells from Whole Slide Images (WSI). The images showed accuracy (99.70%), precision (99.70%), and recall (99.72%) F-Beta (99.63%), and Kappa scores (99.31%), which supersede the scores obtained through principal methodologies [27]. Using the data obtained from the Behavioral Risk Factor Surveillance System (BRFSS) database of the Centers for Disease Control and Prevention, Raghupathiet al., (2012) used neural networks to analyze the combined influence of multiple behavioral habits on chronic diseases. Neural networks have the advantage for analyzing large and complex data sets which herein, makes it possible to identify populations of patients in behavioral risk categories for delivery of wellness programs relating to chronic diseases [28].

### 1.1. Objectives

In this study, we used the multilayer feed-forward back-propagation neural network as one of the popular neural networks in predicting and modeling [29]. For this purpose, we used 80% of the observed data in the training set and the remaining 20% in the model evaluation.

## 2. Methods

This study was descriptive-analytical and cross-sectional research in which the factors related to the stage of change in cervical cancer screening were investigated. The sample populations of this study were female employees of governmental agencies in Birjand, a city located in the east of Iran. The sampling was conducted using a survey and the self-administered questionnaire was completed by 1898 employed women. Inclusion criteria for participation in this study included female employees who were married and aged between 20 and 65 years, with no personal history of cervical cancer, and willingness to participate in the survey. Therefore, we excluded females with a history of hysterectomy or gynecological cancer and women <20 years old and single.

The data were collected using a four-part questionnaire. The first section contains socioeconomic characteristics (age, education, marital status, job, family income, and insurance). The second part of the questionnaire included questions about reproductive and pregnancy characteristics (marriage age, first gestational age, number of children, lactation, and menopause status). The third section of the questionnaire consisted of information about cervical cancer, the history of problems in breasts, and the history of cancer in the close family members, the history of referral for mammography or ultrasound. The four section of the questionnaire consisted of women’s performance and intention regarding screening behaviors of Pap smear.

To evaluate cervical cancer screening behavior stages of change, we used the single item with 3 choices as a stage of behavior changes that was adapted from the mammography stage of change scale described elsewhere [30,31]. Thus, three stages of Pap smear adoption are defined as 1-Pre-contemplation (women who have never had any prior Pap smear and are not planning to get a Pap smear in the coming year) or Relapse (women who have had one or more Pap smears in the previous years but do not intend to have another one in the coming year), 2-Contemplation (women who are planning to get a Pap smear in the coming year, but have not done yet), 3-Action (women who have had a Pap smear with age-specific interval and intent to have another one) or Maintenance (women who have two or more Pap smears on schedule and intend to continue). The content validity of this scale was assessed by summing up the opinions of the expert panel (three health educators, four gynecologists and midwives, two epidemiologists, and one community health nurse). The reliability of this item was also assessed by 20 women who matched with the sample and were out of the target group of this study.

### 2.1. Statistical analysis

In regression analysis, restricted assumptions like normality may be avoided when predicting variables that are categorical such as our study. One of the best ways to overcome this dilemma is using models like ANN models. Since a sufficient sample size was available in the present study, the ANN model seemed to be one of the best analysis methods. The three main components of the neural network are neuron, structure, and weight. The number of input and output layer neurons is determined based on the number of input and response variables. Therefore, the structure of the neural network is determined according to the number of neurons in a hidden layer, the type of connection between neurons in this layer and other layers, and their connection functions [32]. The proper connection function was selected between hyperbolic tangent or sigmoid, to connect the neurons of the input and hidden layers and linear, hyperbolic tangent, SoftMax, or sigmoid functions to connect the neurons of the hidden and output layers in our study [33,34]. Subsequently, we determined the number of optimal neurons in the hidden layer by increasing them to 46 neurons in a network that used the optimal function to connect the neurons of different layers. To avoid the effect of random weight allocation and random correlations, we repeated each grid three times and used their mean sum of square errors as an appropriate indicator in the model evaluation. In the network training, 1519 data were used and the model was confirmed using the remaining 379 data. The performance of the optimal ANN was presented according to the sensitivity, specificity, and accuracy area under the ROC curve (AUC) results. Finally, the importance of input variables including age, education level, job, income level, marriage age, pregnancy, child number, breastfeeding, and menopause, insurance type, disease history (sono and mammography, breast problem, and cancer), and cancer information was determined on Pap smear screening behavior stage of change (output variable). All analyses were performed using Statistical Package for Social Sciences version 22 (SPSS Inc., Chicago, Illinois, USA).

## 3. Results

**Fig. 1:**
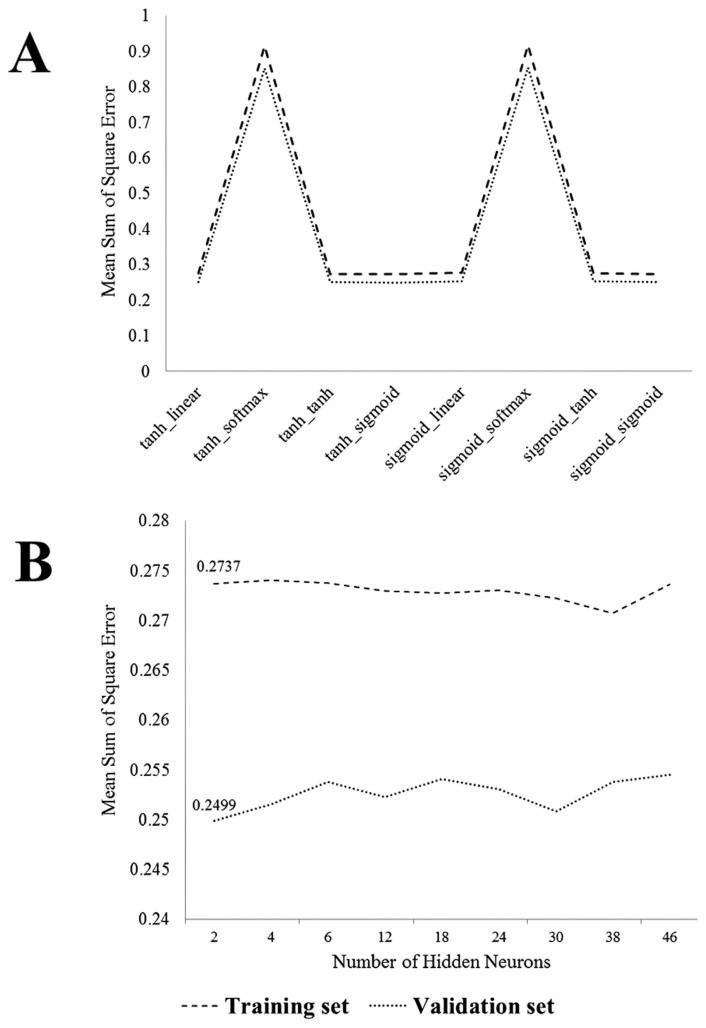
**A)** The mean square error of ANN models in both training and validation phase for different transfer functions; **B)** The mean square error of selected ANN transfer function models in both training and validation phase for different hidden neurons.

**Fig. 2:**
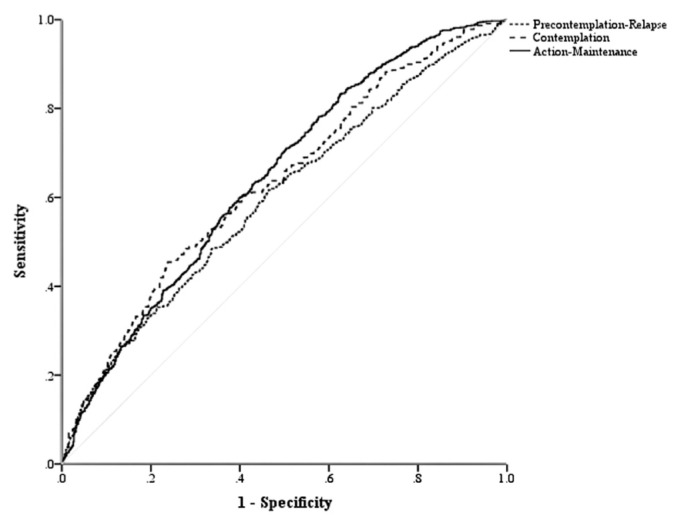
Roc curve of the Artificial Neural Network model.

To determine the best function that can connect different layers of the neural network among the different functions in the connecting input, hidden, and output layers; we examined the results of the neural network with 2 neurons in the hidden layer (Fig. 1A). As can be seen, the neural network with hyperbolic tangent between the neurons of the input and hidden layers and sigmoid function between the neurons of the hidden and output layers had the least mean square error in both the training and validation sets. Furthermore, increasing the number of neurons in the hidden layer did not have an impact on improving the performance of the network (Fig. 1B). Therefore, the neural network with two neurons in the hidden layer and the hyperbolic tangent function for connecting the input-hidden and the sigmoid function for connecting the hidden-output layers with 2.737 e-01 and 2.4996e-01 total mean square errors are optimal in training and validation sets, respectively (Fig. 1). This neural network was able to predict the Pap smear behavior stage of change effectively using these variables (Fig. 2).

As shown in Table 1, 79 participants out of 471 participants and 20 participants out of 113 participants for the precontemplation - Relapse stage in training and validation sets were correctly categorized by the perfect ANNs model. This model also detected 815 participants out of 855 and 223 participants out of 230 participants for the Action-Maintenance stage in training and validation sets by the variables mentioned in the study, but the optimal model was not able to predict women in the contemplation stage correctly. Table 1 also revealed that sensitivity, specificity, and AUC in the validation set were higher than 0.65, 0.43, and 0.59, respectively.

**Fig. 3:**
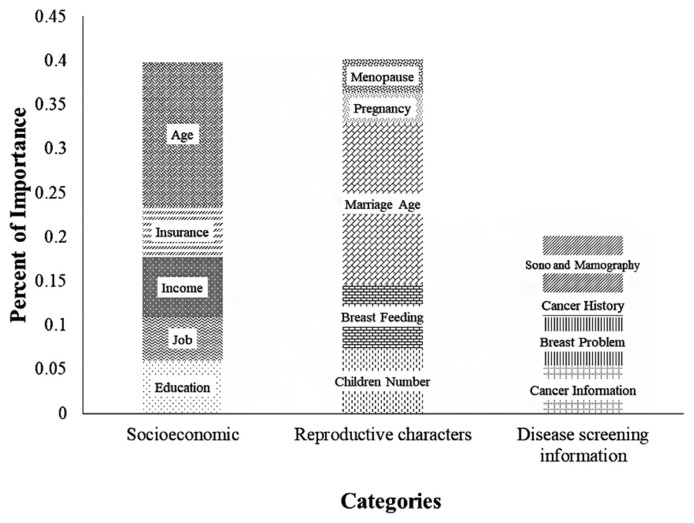
The importance variables from the selected Artificial Neural Network.

The importance of pregnancy characters, socioeconomic factors, and disease screening information were respectively 40.10, 39.80, and 20.10 percent (Fig. 3). Among the variables, marriage age, age, breastfeeding, children number, and education were identified as the most important factors with 18.3, 16.3, 7.3, 7.3, and 7.2 percent, respectively.

As you see in Table 2, women who are 30 years old or younger are often in Precontemplation-Relapse stage and similar stage. Women who are older than 30 years old are often in the Action-Maintenance stage. Women in the Action-Maintenance stage had significantly lower marriage age than other participants. Most mothers that had a baby and experienced breastfeeding were in the Action-Maintenance stage, although about 64 percent of mothers that had no baby and breastfeeding were in Precontemplation-Relapse or Contemplation stage.

## 4. Discussion

ANN models including many different architectures and methods are widely used in many medical applications with better accuracy in results.

Previous studies investigated manual screening methods. Cervigram images in the detection of cervical cancer improved the accuracy of predicting cancer susceptibility, recurrence, and mortality by using ANN models [35–38], however, in this study, ANN was considered as the modeling of predicting Pap smear screening behavior stage.

Our result showed that women who are younger than 30 years of age are often in the Pre-contempltion-relapse stage. Those who are in their 30s or older are often in the action-maintenance stage.

Our result was supported by Tung et al. (2019) who showed more of the older participants aged 40–65 was in a more advanced stage (Action/Maintenance) than younger women and Abbas et al. (2021) who indicated that 67.8% of women were in the Precontemplation Stage of change for performing the Pap smear [39,40]. This is in contrast with the results obtained by Krok-Schoen et al. (2016) which was conducted on 90 women living in Ohio Appalachian and showed that women who are younger than 50 years old are in the action stage in the second visit as well as the results obtained by Studts et al. (2013) who did a study on 345 Appalachian women aged 40–64 years [41,42].

There may be a greater likelihood of obstetric and gynecological problems with aging women who would likely visit more often health clinics and undergo cancer screening. Another possible explanation is that young women are more likely to be unmarried and the gynecological care they receive (Pap test) before marriage may be not be acceptable [43].

Also, people in the action-maintenance stage had significantly lower marriage age than other participants.

Our result is consistent with a community-based cross-sectional study by Aynalem et al. (2020) which was performed on 822 women in the Amhara region, Northwest Ethiopia who had started sexual intercourse at age of 16 and younger and were more likely to utilize cervical cancer screening as compared to those women who had started sexual intercourse after their age of 16 years [44] and it is in contrast with National Survey of Family Growth (NSFG) study stratified by age (15–17, 18–20, 21–29) and sexual activity by Henderson et al. (2013) that showed among females ages 15–17, the proportion screened decreased from 23% to 12% [45]. The possible explanation is that women who start their sexual intercourse earlier have increased lifetime sexual activities and likely experience an increased chance of being infected with sexually transmitted diseases (STD) and abnormal Pap test results which in turn lead to visit health facilities.

Our study indicated that most mothers who had a baby and experienced breastfeeding were in the action-maintenance stage.

This is following a cross-sectional study by **Visanuyothin** et al. (2015) on 675 women aged 30–60 years who found those who had children were more likely to be caregivers. Also, the study conducted by Buki et al. (2007) on 467 Latina women [46,47] and is inconsistent with a qualitative study by Haji Rasul et al. (2016) on 22 women that showed multiple full-term pregnancies did not affect cervical cancer screening [48].

It is explainable that previous contact with reproductive health services in earlier parity of women may increase their awareness about consequences of cervical cancer and they have less fear and embarrassment about undergoing a Pap smear test than the women who have no children. Therefore, the utilization of screening services in the form of gynecological checkups can be directly related to the parity of women [49].

## 5. Conclusion

In conclusion, our findings showed that among many associated variables, the marriage age, age, breastfeeding, and children number were important predictors for the behavioral stage of change. Therefore, it seems to focus on these factors which most of which are modifiable factors, in an appropriate manner in which to influencing cervical cancer screening adherence.

## Figures and Tables

**Table 1:** Power of the different variables for predicting Pap Smear Screening behavior based on triples threshold values in training and validation groups separately.

Actual outcome	Training set (n = 1519)			Validation set (n = 379)			AUC	Sensitivity	Specificity
	
	Precontemplation-Relapse	Contemplation	Action-Maintenance	Precontemplation-Relapse	Contemplation	Action-Maintenance			
Precontemplation-Relapse	79 (16.8%)	0	392	20 (17.7%)	0	93	0.598	0.72	0.48
Contemplation	42	0 (0%)	151	4	0 (0%)	32	0.632	0.67	0.48
Action-Maintenance	40	0	815 (95.3%)	7	0	223 (97%)	0.644	0.69	0.43

**Table 2:** Comparing pap smear behavior stage between different marriage age, age, breast feeding, child number, education, and income categories.

Variables	Precontemplation-Relapse		Contemplation		Action-Maintenance		P-Value
		
	n	%	n	%	n	%	
Age							
20–30	85	46.7	42	23.1	55	30.2	<0.001
30–40	262	28.6	119	13	534	58.4	
40–50	174	28.4	53	8.6	386	63	
≥50	63	33.5	15	8	110	58.5	
Marriage age	23.46 ± 3.64		24.07 ± 4.03		22.95 ± 3.61		<0.001β,γ
Breast feeding							
No	154	44.5	68	19.7	124	35.8	<0.001
Yes	430	27.7	161	10.4	961	61.9	
Child number							
0	138	44.7	65	21	106	34.3	<0.001
1	159	27	88	15	341	58	
2	173	26.7	50	7.7	424	65.5	
3	82	31.2	20	7.6	161	61.2	
≥4	32	35.2	6	6.6	53	58.2	
Education							
Under diploma	25	33.8	3	4.1	46	62.2	0.57
Diploma	57	30.5	22	11.8	108	57.8	
Associate	73	33.2	24	10.9	123	55.9	
Bachelor	338	30.3	146	13.1	631	56.6	
Master or higher	91	30.1	34	11.3	177	58.6	

α significant between Precontemplation-Relapse and Contemplation.

β significant between Precontemplation-Relapse and Action-Maintenance.

γ significant between Contemplation and Action-Maintenance.
